# Loss-of-Function Mutations in *epaR* Confer Resistance to ϕNPV1 Infection in Enterococcus faecalis OG1RF

**DOI:** 10.1128/AAC.00758-18

**Published:** 2018-09-24

**Authors:** Khang Ho, Wenwen Huo, Savannah Pas, Ryan Dao, Kelli L. Palmer

**Affiliations:** a Department of Biological Sciences, The University of Texas at Dallas, Richardson, Texas, USA

**Keywords:** daptomycin, Enterococcus, bacteriophage

## Abstract

Enterococcus faecalis is a Gram-positive opportunistic pathogen that inhabits the human gastrointestinal tract. Because of the high frequency of antibiotic resistance among Enterococcus clinical isolates, interest in using phage to treat enterococcal infections and to decolonize high-risk patients for antibiotic-resistant Enterococcus is rising. Bacteria can evolve phage resistance, but there is little published information on these mechanisms in E. faecalis. In this report, we identified genetic determinants of E. faecalis resistance to phage NPV1 (ϕNPV1). We found that loss-of-function mutations in *epaR* confer ϕNPV1 resistance by blocking phage adsorption. We attribute the inability of the phage to adsorb to the modification or loss of an extracellular polymer in strains with inactivated *epaR*. Phage-resistant *epaR* mutants exhibited increased daptomycin and osmotic stress susceptibilities. Our results demonstrate that *in vitro* spontaneous resistance to ϕNPV1 comes at a cost in E. faecalis OG1RF.

## Abstract (precis)

Enterococcus faecalis is a Gram-positive opportunistic pathogen that inhabits the human gastrointestinal tract. Because of the high frequency of antibiotic resistance among Enterococcus clinical isolates, interest in using phage to treat enterococcal infections and to decolonize high-risk patients for antibiotic-resistant Enterococcus is rising.

## INTRODUCTION

Enterococcus faecalis is a Gram-positive bacterium that inhabits the human gastrointestinal tract and is associated with nosocomial infections (1). Infections caused by E. faecalis can be difficult to treat because of the high frequency of resistance to multiple antibiotics among E. faecalis clinical isolates (2). The antibiotic daptomycin can be used to treat certain infections caused by multidrug-resistant enterococci. Daptomycin is a lipopeptide antibiotic that interacts with the enterococcal cell surface and disrupts membrane structure and function (3).

Bacteriophages (phages) are bacterial viruses and natural predators of bacteria. It is reasonable to expect that phages can be employed to treat bacterial infections. However, phages have not been extensively studied in the Western world in the context of therapeutic application until recently due to the availability of antibiotics (4). In recent years, interest in using phages to treat bacterial infections (phage therapy) has reemerged because of the emergence of multidrug-resistant bacteria. For E. faecalis, promising studies include the use of phage to eliminate biofilm, a major barrier to antibiotic treatment, and to increase survival rates in mouse models of enterococcal infection (5, 6).

One advantage of phage therapy is limited damage to the native microbiome because of the specificity of the phage to its host (7). Typically, lytic phage have narrow host ranges and are species specific or target a range of strains within a species. The first step to a successful phage infection is the attachment of the phage particle to the proper receptor present on the surface of the host cell. Phage receptors have been extensively studied in certain phage families, including the T series phages, Mu, and λ for Gram-negative bacteria (8–11). Some phage receptors have been characterized in Gram-positive bacteria, including receptors for ϕSPP-1 of Bacillus subtilis (12) and the phage c2 group of Lactococcus lactis (13, 14). YueB, the ϕSPP-1 receptor, and phage infection protein (PIP), the phage c2 receptor, are orthologs and are required for irreversible phage adsorption (12). Enterococcal phage receptors have not been well characterized. Previously, we and collaborators identified PIP as a receptor and potential DNA channel for the E. faecalis phages ϕVPE25 and ϕVFW (15).

Bacteria can evolve phage resistance. Mechanisms of phage resistance include modification or loss of the phage receptor (16). However, as phage receptors generally serve physiological functions in the cell, the modification or loss of a receptor could come at a cost for the bacterial host. For example, spontaneous phage-resistant mutants have altered antibiotic sensitivity in Pseudomonas aeruginosa (17). Phages utilizing receptors that have roles in antibiotic resistance could be advantageous for resensitizing resistant bacteria to antibiotics.

Considering the increasingly limited treatment options for E. faecalis infections and the revival of interest in using phage therapy to treat bacterial infections, it is crucial that we know the receptor(s) of enterococcal phages, since effective phage cocktails use phages targeting multiple different receptors (18). Moreover, the roles of these receptors in enterococcal physiology should be elucidated. The tailed virulent phage NPV1 (ϕNPV1) was found in previous studies to infect E. faecalis OG1RF (19, 20). In this study, we used a combination of genomic and genetic approaches to investigate the ϕNPV1 receptor in E. faecalis OG1RF.

## RESULTS

### Deletion of *epaR* alters susceptibility to ϕNPV1.

**FIG 1:**
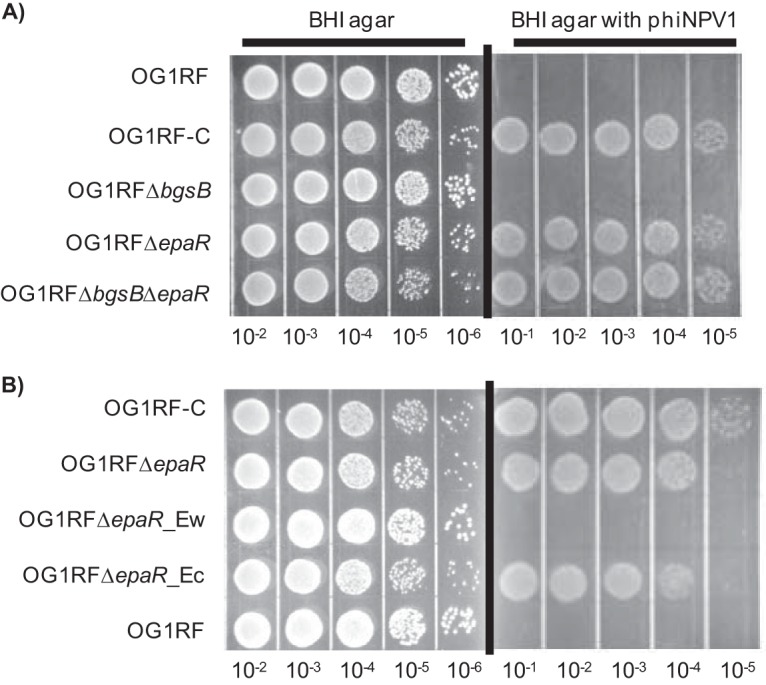
Phage susceptibility assays. Overnight cultures were diluted in phosphate-buffered saline (PBS) and spotted on BHI plates with or without 10^9^ PFU/ml ϕNPV1. Images were taken after 18 h of incubation at 37°C. The images shown are representative of three independent trials. (A) ϕNPV1 susceptibility of E. faecalis OG1RF and derivatives. (B) ϕNPV1 susceptibility of complemented strains of OG1RF Δ*epaR*.

We isolated a OG1RF ΔPIP (15) strain with spontaneous resistance to ϕNPV1 (Fig. 1). We refer to this strain as OG1RF-C. The genome sequence of OG1RF-C was determined. We identified nonsynonymous substitutions in *epaR*, *bgsB*, *iolA2*, and OG1RF_10252 (Table 1). *epaR* is one of the 18 conserved genes of the *epa* gene cluster (*epaA-epaR*), which codes for synthesis of the enterococcal polysaccharide antigen (Epa) (20). *epaR* encodes a putative glycosyltransferase with 5 predicted transmembrane domains, and its role in Epa biosynthesis has not been investigated. The product of *bgsB* is a putative cytoplasmic protein catalyzing the transfer of glucose from UDP-glucose to diacylglycerol (DAG) to form monoglucosyl-DAG. An additional glucose is added to glucosyl-DAG by *bgsA*, forming diglucosyl-DAG. From diglucosyl-DAG, the polymerization of glycerol phosphate can occur, resulting in lipoteichoic acid (LTA) (21). *iolA2* is predicted to encode a methylmalonate-semialdehyde dehydrogenase, which catalyzes the breakdown of malonic semialdehyde to acetyl-coenzyme A (acetyl-CoA) and CO_2_ (22). OG1RF_10252 is predicted to encode an acyl-ACP_TE domain (pfam01643; E value = 7.510e^−114^) which catalyzes the termination of fatty acyl group extension by hydrolyzing an acyl group on the fatty acid.

**TABLE 1:** SNPs detected in OG1RF-C strain^*a*^

Position	bp change	Amino acid change	Annotation
1800903	C→T	D361N	*epaR*
1878245	G→T	S453Y	*bgsB*
2308650	C→A	G134*	*iolA*
260665	Insertion of A	H44fs	OG1RF_10252

a SNPs, single-nucleotide polymorphisms; * indicates a stop codon.

To begin to elucidate the roles of these genes in ϕNPV1 susceptibility, we constructed in-frame deletions of *epaR* and *bgsB*, generating strains OG1RF Δ*epaR* and OG1RF Δ*bgsB*, respectively, and a double-deletion strain, OG1RF Δ*epaR* Δ*bgsB*. The phage susceptibilities of each of these mutants were assayed. The deletion of *epaR* alone was sufficient to confer phage resistance (Fig. 1). In contrast, the deletion of *bgsB* alone did not alter phage susceptibility. These results indicate that variation in *epaR* is the major factor conferring resistance to ϕNPV1 in OG1RF-C. Since we observed that the deletion of *epaR* in OG1RF conferred phage resistance to the same extent as that observed for OG1RF-C, we did not investigate the effects of *iolA2* and OG1RF_10252 on phage resistance in this study.

### The OG1RF-C *epaR* allele confers ϕNPV1 resistance.

To determine whether *epaR* mutation is the major contributor to ϕNPV1 resistance in OG1RF-C, we generated strain OG1RF Δ*epaR_Ec*, an OG1RF Δ*epaR* strain complemented in *cis* with the *epaR* allele from OG1RF-C. We also generated strain OG1RF Δ*epaR_Ew*, an OG1RF Δ*epaR* strain with a reconstituted wild-type *epaR*. Complementation with the *epaR* allele of OG1RF-C conferred phage resistance to OG1RF Δ*epaR* (Fig. 1B). The wild-type *epaR* allele restored phage susceptibility to OG1RF Δ*epaR* (Fig. 1B). Because the mutated *epaR* allele from OG1RF-C confers a phage resistance phenotype, as did the deletion of *epaR*, we infer that the *epaR* mutation in OG1RF-C confers loss of function.

### Growth rates of E. faecalis strains.

We determined generation times for wild-type E. faecalis OG1RF, OG1RF-C, and the *epaR* and *bgsB* deletion mutants and complemented strains cultured in brain heart infusion (BHI) broth (see Table S1 in the supplemental material). The average generation times ranged from a minimum of 27.8 min for the *bgsB* deletion mutant to a maximum of 42.7 min for the *epaR bgsB* double-deletion mutant. The generation times for the wild-type and OG1RF-C strains were similar (30.4 versus 33.9 min, respectively).

### *epaR* is required for phage adsorption.

**FIG 2:**
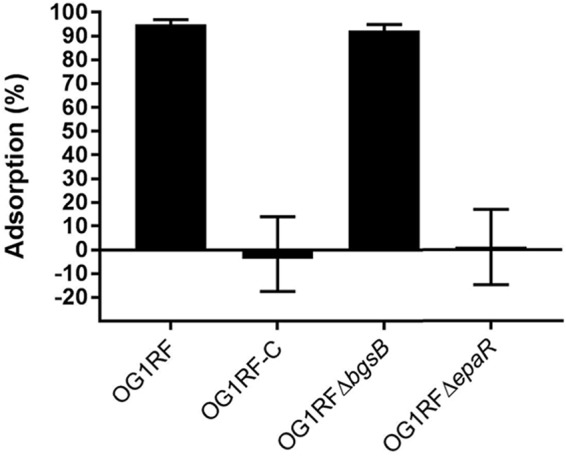
ϕNPV1 adsorption assays. Overnight cultures were diluted 1:5 in fresh BHI and equilibrated at 37°C. ϕNPV1 was added at an MOI of 10^−2^. After 15 min of incubation, 1 ml of each culture was centrifuged, and the titer of the supernatant was determined with the phage spot assay. A medium with only phage was used as the control. Percent adsorption was calculated as [(PFU culture − PFU control)/PFU control] × 100. The data are the average of the results from three independent trials.

We were next interested in how *epaR* inactivation protects OG1RF from ϕNPV1 infection. After 15 min of incubation with ϕNPV1, ∼95% of the phage adsorbed to wild-type OG1RF (Fig. 2). In contrast, under the same conditions, ∼1 to 2% of the phage adsorbed to OG1RF-C or OG1RF Δ*epaR*. Consistent with the observation that *bgsB* deletion alone confers no significant phage resistance, ∼90% of the phage adsorbed to OG1RF Δ*bgsB* within the same experimental settings. These data indicate that *epaR* is required for ϕNPV1 adsorption.

### Inactivation of *epaR* alters the Epa polymer.

**FIG 3:**
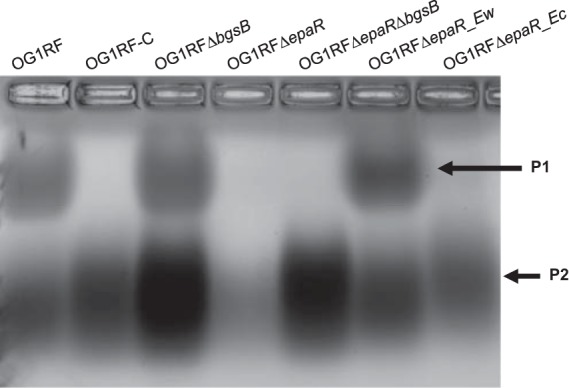
Carbohydrate extract analysis. Carbohydrate was extracted from 200 ml overnight cultures and visualized with Stains-all. The image shown is representative of two independent trials.

Next, we sought to determine whether the Epa polymer was altered in mutants defective for ϕNPV1 adsorption. The Epa polymer has been extracted and visualized by different groups using different methods (20, 23). We based our method on that from Teng et al. (20). We found that solubilizing the precipitation with 50% acetic acid improved visualization of the polymers. Gel electrophoresis analysis of carbohydrate extracts found that OG1RF with either an *epaR* deletion or the *epaR* allele from OG1RF-C exhibited the loss of a band (P1) that is present in wild-type OG1RF, OG1RF Δ*bgsB*, and the reconstituted *epaR* strain, OG1RF Δ*epaR_Ew* (Fig. 3). We conclude that P1 represents an *epaR*-dependent polymer. Note that the cationic dye Stains-All was used for polymer detection. With the staining methodology used, we cannot state conclusively whether the P1 polymer fails to be synthesized in *epaR* mutants or if it is synthesized but has a different charge than in wild-type OG1RF. Since ϕNPV1 cannot bind to *epaR* mutants, we hypothesized that the P1 polymer is the phage receptor. However, when we preincubated ϕNPV1 with crude carbohydrate extract prior to the infection of host cells, we did not observe a decrease in PFU for any extract (Fig. S1), suggesting that the polymers in the crude extract are not sufficient for phage adsorption.

We observed an increase in the intensity of the P2 band in OG1RF Δ*bgsB* and OG1RF Δ*bgsB* Δ*epaR* compared to that in wild-type OG1RF, suggesting that the product P2 is increased in these two deletion strains. Since the deletion of *bgsB* in E. faecalis results in an accumulation of LTA (21), product P2 may represent LTA.

### ϕNPV1-resistant mutants have increased susceptibility to daptomycin.

**FIG 4:**
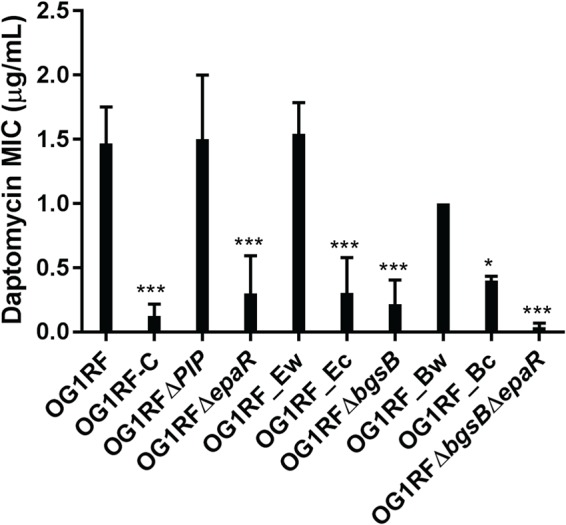
Daptomycin MICs of E. faecalis OG1RF and derivatives. Daptomycin MIC was determined by Etest. Data are the average of the results from at least three independent trials. For statistical analysis, daptomycin (DAP) MICs were compared to that of wild-type OG1RF. ***, *P* < 0.001; *, *P* < 0.05. Note that the names of complemented strains (see Table 3) have been shortened for clarity.

Dale et al. reported increased daptomycin susceptibility in an OG1RF derivative with a deletion in *epaO* (23). Moreover, we identified a *bgsB* mutation in a laboratory-evolved Enterococcus faecium isolate with decreased daptomycin susceptibility (24). Because of these results, we investigated the daptomycin susceptibilities of our OG1RF mutants (Fig. 4). We found that OG1RF-C, OG1RF Δ*epaR*, and OG1RF Δ*epaR* complemented with the OG1RF-C *epaR* allele were each significantly more susceptible to daptomycin than OG1RF. Interestingly, the deletion of *bgsB* also conferred increased daptomycin susceptibility (Fig. 4). This was complemented by the expression of the wild-type *bgsB* allele in *cis* but not by the expression of the OG1RF-C *bgsB* allele in *cis* (Fig. 4). Finally, daptomycin susceptibility was substantially altered in the OG1RF Δ*epaR* Δ*bgsB* mutant, with 3 of 6 experimental trials resulting in an MIC below the level of detection of the Etest strip (<0.016 μg/ml; a value of 0.008 μg/ml was used for these data points in statistical analysis). Without complete data regarding the MIC of OG1RF Δ*epaR* Δ*bgsB*, we did not quantitatively determine whether there is a synergistic relationship between *bgsB* and *epaR* regarding daptomycin susceptibility.

### *epaR* mutants have increased sodium chloride stress susceptibility.

**FIG 5:**
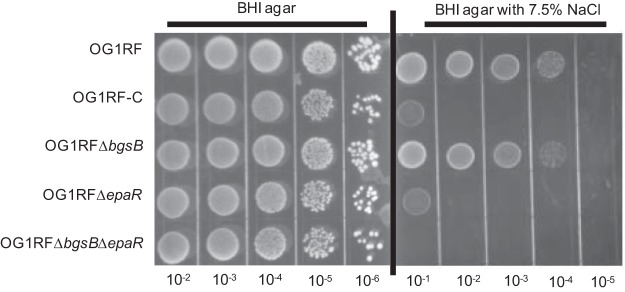
Susceptibility to sodium chloride-induced osmotic stress. Overnight cultures were diluted in PBS and spotted on BHI plates with or without sodium chloride. Images were taken after 72 h of incubation. The image shown is representative of three independent trials.

The *epa* gene cluster was upregulated when E. faecalis V583 was grown with 6.5% sodium chloride supplementation, indicating that the Epa polymer has a role in the osmotic stress response (25). As such, we investigated the effect of sodium chloride on our *epaR* mutants. We tested our mutants for their tolerance for sodium chloride stress using BHI agar supplemented with sodium chloride at concentrations of 0%, 2.5%, 5%, and 7.5%. Overnight cultures in stationary phase were serially diluted and spotted on these agars. We observed fewer CFU for OG1RF-C, OG1RF Δ*epaR*, and OG1RF Δ*epaR* Δ*bgsB* than for the wild type at 7.5% sodium chloride after 72 h of incubation (Fig. 5). When sodium chloride concentrations of 5% or lower were used, no effect on growth was observed.

### Multiple different spontaneous *epaR* mutations confer ϕNPV1 resistance.

We isolated 9 spontaneous ϕNPV1-resistant mutants of OG1RF and sequenced the *epaR* region of these mutants. All 9 mutants have nonsynonymous substitutions in *epaR* (Table 2). Since we know that mutations in *epaR* affect daptomycin susceptibility, we also determined the daptomycin MIC of these ϕNPV1-resistant strains and found that all were more significantly more susceptible to daptomycin than was the wild type (Fig. S2).

**TABLE 2:** *epaR* variations in spontaneous ϕNPV1-resistant E. faecalis OG1RF strains

Strain	Mutation type	Mutation	Amino acid change^*a*^
R3	Transversion	C→A	G409V
R5		C→A	D428Y
R7		C→A	G409V
R10		C→A	G409V
R8		C→A	D428Y
R4		T→A	K424*
R9	Transition	C→T	G378D
OG1RF-C		C→T	D361N
R6	Deletion	Deletion of 101 bp	G372fs
R2		Deletion of 1 bp	D373fs

a fs, frameshift; * indicates a stop codon.

Most of the EpaR sequence (amino acid positions 35 to 458 of 484 total) is a predicted sugar transferase domain (TIGR03025; E value = 2.45e^−137^). This domain consists of a conserved C-terminal region responsible for the sugar transferase activity (pfam02397) and a variable N-terminal region with predicted flippase activity. All *epaR* mutations in spontaneously ϕNPV1-resistant OG1RF strains occur in the region encoding the sugar transferase domain (Table 2). We identified other proteins containing the same predicted sugar transferase domain as EpaR, and we determined that the altered amino acid positions in our mutants are conserved across most of these proteins (Fig. S3).

## DISCUSSION

Due to the high frequency of antibiotic resistance in E. faecalis, alternatives to antibiotics, such as phage therapy, are of increasing interest in the United States. In this study, we investigated mechanisms for spontaneous phage resistance in E. faecalis. We have reported here that *epaR* is indispensable for ϕNPV1 adsorption to E. faecalis OG1RF and that inactivating mutations in *epaR* constitute a major pathway for ϕNPV1 resistance in this strain background. We also found that inactivating mutations in *epaR* and *bgsB* resulted in increased susceptibilities to daptomycin and sodium chloride stress. Our results show that resistance to ϕNPV1 comes at a cost.

Adsorption to the host is the first step to a productive phage infection. For a tailed phage particle to successfully adsorb to the host, the tail apparatus on the phage must recognize the corresponding receptor(s) on the host cell surface. When challenged with a high phage titer in a resource-limited environment, receptor mutations in host cells are favored over the use of intracellular defense mechanisms (26). This preference for receptor mutations is an especially important consideration in the design of phage cocktails, as using phage that recognize the same receptors could result in decreased efficacy of the treatment (18). Receptors for enterococcal phages have not been well studied. We and collaborators recently identified PIP as a receptor for phages ϕVPE25 and ϕVFW, but PIP is not the sole player in host cell recognition, as ϕVFW and ϕVPE25 can still adsorb to a PIP deletion strain (15). PIP may act as a DNA channel, as is implicated in studies of L. lactis (14).

The *epa* gene cluster is involved in the synthesis of a cell wall rhamnose polysaccharide referred to as Epa. There is precedence for cell wall rhamnose polysaccharides being phage receptors. The structure of the rhamnose polysaccharide dictates phage host range in L. lactis and Streptococcus mutans (27, 28). In E. faecalis, the *epa* gene cluster consists of 18 core genes (*epaA* to *epaR*) and a set of strain-variable genes that occur downstream (20, 29). Unfortunately, the Epa structure has not been determined (30), which is a critical gap in knowledge about the enterococcal cell surface.

The connection between the *epa* gene cluster and ϕNPV1 resistance was first investigated by Teng et al., who assessed ϕNPV1 susceptibilities of E. faecalis OG1RF mutants with disruptions in *epaA*, *epaB*, *epaE*, *epaM*, and *epaN* (20). No ϕNPV1 plaques were obtained for *epaB*, *epaE*, *epaM*, and *epaN* mutants, and plaque production was reduced by 50% in the *epaA* mutant compared to that in the wild type. However, when the wild-type strain and the *epaA* and *epaB* mutants were assessed for ϕNPV1 adsorption, no differences were noted. Teng et al. also examined the polysaccharide content of their mutants and found that production of the P1 product was absent in the *epaB*, *epaM*, *epaN*, and *epaE* mutants, but a new polysaccharide product referred to as PS12 was synthesized. For the *epaA* mutant, both P1 and P12 were produced. The results from the study by Teng et al. suggest that a complete Epa product is required for productive ϕNPV1 infection. Our results support this conclusion, as ϕNPV1 did not adsorb to our *epaR* mutants, nor did *epaR* mutants synthesize the P1 (or P12) product. However, the P1 product may not be the only requirement for ϕNPV1 adsorption because no significant decrease in PFU was observed when ϕNPV1 was preincubated with crude polysaccharide extracts from OG1RF strains with either wild-type or mutant *epaR* (Fig. S1). Alternatively, the availability of P1 to the phage may differ in whole cells versus crude extracts.

We investigated the daptomycin susceptibilities of our ϕNPV1-resistant strains with *epaR* mutations because a mutation elsewhere in the *epa* locus was previously linked to daptomycin susceptibility in E. faecalis. Specifically, Dale et al. reported that the deletion of *epaO* results in increased daptomycin susceptibility (23). Daptomycin is a lipopeptide antibiotic that is used to treat certain Gram-positive bacterial infections (31). The mechanism of action for daptomycin in B. subtilis begins with daptomycin binding to the cell membrane and ultimately leads to the displacement of membrane-associated proteins essential for cell wall and phospholipid biosynthesis (32). In our study, we found that inactivating mutations in *epaR* lead to increased daptomycin susceptibility in E. faecalis. The loss of the Epa polymer results in defects in cell wall architecture (20, 33), suggesting that this polymer plays a critical role in enterococcal cell surface physiology. More research on the Epa polymer is required to mechanistically assess its contribution to antibiotic susceptibility in enterococci. In our study, the deletion of *bgsB* also resulted in increased daptomycin susceptibility in E. faecalis. The deletion of *bgsB* results in a loss of glycolipids in the membrane, a longer chain length in the LTA, and increased charge density of the membrane (21). A higher charge density might contribute to daptomycin susceptibility through charge-charge interaction with the calcium-bound daptomycin, but this is speculative. Note that a weakness of our study is that we did not evaluate whether susceptibilities to other antibiotics are altered concomitantly with spontaneous ϕNPV1 resistance or as a result of *epaR* or *bgsB* deletion. Therefore, we cannot comment on whether the altered antibiotic susceptibilities of these strains are specific to daptomycin or are a general defect potentially related to altered membrane/cell wall permeability.

In summary, in this study, we characterized a mechanism for spontaneous ϕNPV1 resistance in E. faecalis OG1RF and demonstrated that *in vitro* spontaneous ϕNPV1 resistance is accompanied by fitness trade-offs, including altered susceptibilities to an antibiotic and to osmotic stress. Experiments for future work include determining the host range of ϕNPV1 and whether other enterococcal phage use the Epa polymer as a receptor for E. faecalis adsorption. A critical experiment in terms of possible therapeutic application of ϕNPV1 is to determine whether ϕNPV1 resistance arises *in vivo* (i.e., in the gastrointestinal tract, or during experimental treatment of an E. faecalis infection using phage therapy) by the same mechanism as that *in vitro*. If ϕNPV1 resistance arises *in vivo* by *epaR* or other *epa* locus inactivation and this confers an *in vivo* fitness cost to E. faecalis, ϕNPV1 and/or other Epa-targeting phages could be of utility for anti-E. faecalis therapies.

## MATERIALS AND METHODS

### Bacterial strains, media, and bacteriophages.

A complete list of the bacterial strains and bacteriophage used in this study can be found in Table 3. E. faecalis strains were cultured in brain heart infusion (BHI) at 37°C without agitation. Escherichia coli strains were cultured in LB broth at 37°C with shaking at 225 rpm, unless otherwise stated. Plates of the appropriate media were made by adding 1.5% agar to the broth prior to autoclaving. For MIC testing, Muller-Hinton medium supplemented with 1.5% agar (MHA) was used. Phages were stored in phage buffer, as previously described (34). Chloramphenicol (Cm) was used at a concentration of 15 μg/ml when required for selection. 5-Bromo 4-chloro-3-indolyl-β-d-galactopyranoside (X-Gal) was used at 120 μg/ml and 40 μg/ml for E. faecalis and E. coli, respectively. The upper soft agar for phage assays was M17 medium supplemented with 0.75% agar, while the lower layer was BHI supplemented with 1.5% agar.

**TABLE 3:** Strains and plasmids used in this study

Strain or plasmid	Description	Reference or source
Strains		
E. faecalis		
OG1RF	Human oral cavity isolate	40, 41
OG1RF ΔPIP	PIP deletion strain	15
OG1RF-C	OG1RF ΔPIP ϕNPV1-resistant strain	This work
OG1RF Δ*bgsB*	OG1RF *bgsB* deletion mutant	This work
OG1RF Δ*epaR*	OG1RF *epaR* deletion mutant	This work
OG1RF Δ*bgsB* Δ*epaR*	OG1RF *bgsB* and *epaR* double-deletion mutant	This work
OG1RF Δ*epaR_Ew*	OG1RF Δ*epaR* with wild-type *epaR* complementation in *cis*	This work
OG1RF Δ*epaR_Ec*	OG1RF Δ*epaR* complemented with OG1RF-C *epaR* allele in *cis*	This work
OG1RF Δ*bgsB_Bw*	OG1RF Δ*bgsB* with wild-type *bgsB* complementation in *cis*	This work
OG1RF Δ*bgsB_Bc*	OG1RF Δ*bgsB* complemented with OG1RF-C *bgsB* allele in *cis*	This work
OG1RF_R2	OG1RF ϕNPV1-resistant strain	This work
OG1RF_R3	OG1RF ϕNPV1-resistant strain	This work
OG1RF_R4	OG1RF ϕNPV1-resistant strain	This work
OG1RF_R5	OG1RF ϕNPV1-resistant strain	This work
OG1RF_R6	OG1RF ϕNPV1-resistant strain	This work
OG1RF_R7	OG1RF ϕNPV1-resistant strain	This work
OG1RF_R8	OG1RF ϕNPV1-resistant strain	This work
OG1RF_R9	OG1RF ϕNPV1-resistant strain	This work
OG1RF_R10	OG1RF ϕNPV1-resistant strain	This work
E. coli		
EC1000	Cloning host, provides *repA* in *trans*	42
Plasmids		
pLT06	Cloning vector, temp-sensitive *repA*, Cm^r^^*a*^	36
pLT06_Δ*bgsB*	*bgsB* deletion construct	This work
pLT06_Δ*epaR*	*epaR* deletion construct	This work
pLT06_Ew	*epaR* wild-type allele complementation construct	This work
pLT06_Ec	*epaR* OG1RF-C allele complementation construct	This work
pLT06_Bw	*bgsB* wild-type allele complementation construct	This work
pLT06_Bc	*bgsB* OG1RF-C allele complementation construct	This work

a Cm^r^, chloramphenicol resistant.

### Routine molecular techniques and DNA sequencing.

Routine PCRs were performed using *Taq* polymerase (NEB), as per the manufacturer's instructions. Phusion polymerase (Fisher) was used for cloning procedures, as per the manufacturer's instructions. Plasmid was purified using the GeneJET plasmid miniprep kit (Fisher). Genomic DNA was isolated using the UltraClean microbial DNA isolation kit (Mo Bio). Restriction enzymes, Klenow fragment, T4 polynucleotide kinase (PNK), T4 DNA ligase, and calf intestinal phosphatase (CIP) from NEB were used as instructed by the manufacturer. DNA sequencing was performed at the Massachusetts General Hospital DNA sequencing facility. A complete list of the primers used in this study can be found in Table S2.

### Growth curves.

Growth curves were performed in triplicate with a BioTek Synergy microplate reader, essentially as previously described (35). Overnight broth cultures were diluted 1:1,000 into fresh BHI broth and aliquoted into 96-well plates. The optical density at 600 nm of cultures was monitored for 20 h.

### Phage spot assay.

An exponentially growing culture (0.5 ml) was added to 3 ml soft agar and poured onto BHI agar. Ten microliters of the phage mixture of which the titer was to be determined was spotted onto the soft agar. Plaques were counted after 16 h of incubation at 37°C, unless otherwise stated.

### Phage propagation and storage.

Phage stocks were prepared by mixing 450 μl of an overnight culture of E. faecalis OG1RF with ϕNPV1 at a multiplicity of infection (MOI) of 10^−2^. The mixture was incubated at 37°C for 15 min and subsequently added to 3 ml M17 soft agar maintained at 55°C. The soft agar was then poured onto BHI agar and incubated at 37°C for 18 h. Five milliliters of phage buffer was added to the confluently lysed plate and incubated for 20 min at 37°C with shaking at 75 rpm. The lysate was then collected and centrifuged at 16.6 × *g* for 1 min to remove cellular debris. The supernatant was filtered with a Whatman 0.2-μm filter to obtain the phage stock. The phage stock was stored at 4°C in the dark. Phage titer was determined using phage spot assays.

### Generation of OG1RF deletion mutants.

Gene deletion was carried out via the markerless deletion procedure described by Thurlow et al. (36), with some modifications. Briefly, two 1.0-kb regions flanking *epaR* were amplified with primers 1 to 4 from Table S2. The two amplified products were ligated with an overlap PCR extension through a 21-bp complementary region underlined in Table S2. The approximately 2.0-kb product was purified and digested with BamHI and EcoRI. The digested product was ligated to plasmid pLT06 through restriction sites added on the primers (highlighted in red in Table S2). The ligation product was then purified and electroporated and propagated in E. coli EC1000. OG1RF was made electrocompetent using the glycine method (3% glycine) (37) and transformed with 1 μg of the plasmid. OG1RF transformants were screened for successful transformation and subsequently inoculated in BHI supplemented with Cm at 30°C. The culture was diluted 1:100 in BHI and incubated at 30°C for 2 h, followed by 42°C for 4 h. Dilutions of the culture were plated on BHI agar supplemented with Cm and X-Gal, and large blue colonies were screened for plasmid integration using primers 5 and 19. The positive colonies were then restruck, incubated at 42°C, and screened once again for plasmid integration. Positive clones were cultured in BHI broth at 30°C for 18 h. To counterselect against clones harboring the plasmid, dilutions of the culture were made on MM9YEG agar, and the deletion of *epaR* was determined by colony PCR with primers 5 and 6 after 36 h of incubation at 37°C. Clones positive for the deletion were then restruck on BHI agar and screened again using the same primers. Positive clones were verified for plasmid loss by streaking on BHI agar supplemented with Cm. The *epaR* region was sequenced to confirm the deletion. The deletion of *bgsB* was obtained in a similar fashion.

### Complementation.

Complementation of *epaR* in an OG1RF Δ*epaR* background was obtained using a strategy similar to that with deletion. The insert containing the *epaR* gene and 500-bp upstream and downstream flanking regions were amplified from either OG1RF or OG1RF-C using primers 7 and 8. pLT06 was digested with SphI and blunt-ended with Klenow fragment; the blunt-end product was then treated with CIP. The insert was phosphorylated with T4 PNK and blunt-end ligated to pLT06. The plasmid was purified and transformed into EC1000. Clones with the correct insert size were screened and their plasmids isolated. Subsequent steps for transformation of OG1RF, integration of the plasmid, and counterselection on MM9YEG were as described above for the deletion process. Positive clones for the complementation were confirmed with primers 5 and 6 after counterselection on MM9YEG. The complemented *epaR* allele was verified through Sanger sequencing.

### Assessment of phage resistance.

For the assessment of ϕNPV1 resistance, 500 μl of an 8 × 10^9^ PFU ϕNPV1 stock was added to 3 ml M17 soft agar. The mixture was then poured onto BHI agar. Bacterial culture dilutions were spotted on the soft agar and incubated at 37°C for 18 h. ϕNPV1-resistant bacteria grow on ϕNPV1-containing agar. ϕNPV1-susceptible bacteria do not grow on ϕNPV1-containing agar.

### Assessment of sodium chloride stress tolerance.

For an assessment of osmotic stress tolerance, BHI plates were supplemented with NaCl (0%, 2.5%, 5%, and 7.5%). Overnight cultures of bacteria were serially diluted and spotted on NaCl-supplemented plates. The plates were imaged after 72 h of incubation.

### Phage adsorption assay.

An overnight bacterial culture was diluted 1:5 in fresh BHI broth. The culture was then equilibrated at 37°C for 20 min in a water bath. ϕNPV1 was added at an MOI of 10^−3^. After 15 min, a 1-ml aliquot was centrifuged at 16.6 × *g* for 1 min at room temperature. Five microliters of the supernatant was collected, and its titer was determined with the phage spot assay. A medium with only phage added (no bacteria) was used as a control. Percent adsorption was determined as follows:

(1) $$\text{percent adsorption}=\frac{{\text{PFU}}_{\text{control}}-{\text{PFU}}_{\text{supernatant}}}{{\text{PFU}}_{\text{control}}}\times 100$$

### Isolation of ϕNPV1-resistant mutants.

For isolation of a ϕNPV1-resistant strain from an OG1RF ΔPIP background, ϕNPV1 was used to infect OG1RF ΔPIP in a soft agar overlay. The confluently lysed plates were incubated until presumptive phage-resistant colonies arising in the soft agar were observed. These colonies were cultured in BHI broth and used as hosts for ϕNPV1 infection to confirm phage resistance. A confirmed ϕNPV1-resistant strain, referred to in our study as OG1RF-C, was stocked and used for genome sequencing.

For the isolation of ϕNPV1-resistant strains from an OG1RF background, 500 μl of an overnight culture of OG1RF was infected with ϕNPV1 at an MOI of 10^−1^ in a soft agar overlay. Ten colonies that arose on the confluently lysed plate were struck on BHI plates and incubated at 37°C for 18 h. Single colonies from each of the plates were tested for phage resistance by cross-streaking against ϕNPV1. Nine colonies that showed little to no lysis were stocked and used for daptomycin susceptibility testing and *epaR* sequencing.

### Polysaccharide analysis.

Polysaccharide extraction was performed as described by Teng et al., with some modifications (20). Two hundred milliliters of an overnight culture was centrifuged and resuspended in 750 μl of 50 mM Tris buffer (pH 7.5). Mutanolysin (0.25 U/μl) and lysozyme (5 mg/ml) were added to the suspension. The suspension was incubated at 37°C for 2 h. Subsequently, 10 mM MgSO_4_, 2.5 mM CaCl_2_, 0.15 mg/ml DNase I, and 0.15 mg/ml RNase A were added. After an additional 2 h of incubation at 37°C, the suspension was centrifuged and the cellular debris discarded. Proteinase K (100 μg/ml) was added to the clear supernatant, and the mixture was incubated for 16 h. Afterwards, the supernatant was extracted twice with chloroform-phenol-isoamyl alcohol (Sigma-Aldrich) and once with chloroform. Ethanol was added to a final concentration of 80% to precipitate the polysaccharide. The precipitate was collected by centrifugation and air-dried. The pellet was resuspended in 50% acetic acid (vol/vol) in deionized water, and the insoluble material was removed by centrifugation. Thirty microliters was loaded onto a 1% agarose gel and electrophoresed for 30 min at 130 V. The gel was soaked in staining solution containing Stains-All (Alfa Aesar) and left overnight with gentle rocking. The staining solution was 25% isopropanol, 10% formamide, 65% water, and 0.005% Stains-All. After 18 h, the gel was destained under light for 40 min prior to visualization.

### Daptomycin MIC.

Daptomycin MIC was assessed using Etest strips (bioMérieux). Three to five colonies of similar sizes were resuspended in 500 μl BHI broth and distributed evenly over an MHA plate using a sterile cotton swab. Please note that this inoculation method deviates from clinical susceptibility testing criteria in that we did not determine the CFU of our inocula nor normalize the density of the inocula to a McFarland standard. A daptomycin Etest strip was placed onto the plate, and the plate was incubated for 18 h at 37°C. The MIC was determined by recording the number closest to the zone of inhibition. The MIC reported for each strain is the average of at least three independent trials. For trials in which the daptomycin MIC was below the detection limit of the strip (<0.016 μg/ml), the MIC was reported as 0.008 μg/ml for the purposes of statistical analysis. Data were analyzed using the two-tailed unpaired Student's *t* test.

### Whole-genome sequencing and analysis of OG1RF-C.

OG1RF-C genomic DNA was isolated from overnight broth culture using the UltraClean Microbial DNA isolation kit (Mo Bio), as per the manufacturer's instruction. The genomic DNA (gDNA) was sequenced using a MiSeq platform with 2 × 150-bp chemistry at MR DNA (Shallowater, TX). After sequencing, the reads were mapped to the complete OG1RF reference (RefSeq accession no. NC_017316.1) using CLC Genomics Workbench (Qiagen). Putative mutations were detected using the basic variant detector in CLC Genomics Workbench. Variants occurring at ≥50% frequency in the read assembly and resulting in nonsynonymous substitutions were confirmed with Sanger sequencing. BLASTP and NCBI Conserved Domains were used to analyze conserved domains in proteins. Amino acid alignment was performed with ClustalW (38). Transmembrane helices were predicted with TMHMM version 2.0 (39).

### Data availability.

The Illumina reads for OG1RF-C have been deposited in the Sequence Read Archive under the accession number PRJNA450206.

## Supplementary Material

Supplemental file 1

## References

[1] Lebreton F, Willems RJL, Gilmore MS 2014 Enterococcus diversity, origins in nature, and gut colonization. *In* Gilmore MS, Clewell DB, Ike Y, Shankar N (ed), Enterococci: from commensals to leading causes of drug resistant infection. Massachusetts Eye and Ear Infirmary, Boston, MA. 24649513

[2] Kristich CJ, Rice LB, Arias CA 2014 Enterococcal infection–treatment and antibiotic resistance. *In* Gilmore MS, Clewell DB, Ike Y, Shankar N (ed), Enterococci: from commensals to leading causes of drug resistant infection. Massachusetts Eye and Ear Infirmary, Boston, MA. 24649510

[3] Tran TT, Munita JM, Arias CA 2015 Mechanisms of drug resistance: daptomycin resistance. Ann N Y Acad Sci 1354:32–53. doi:10.1111/nyas.12948. 26495887 PMC4966536

[4] Deresinski S 2009 Bacteriophage therapy: exploiting smaller fleas. Clin Infect Dis 48:1096–1101. doi:10.1086/597405. 19275495

[5] Khalifa L, Brosh Y, Gelman D, Coppenhagen-Glazer S, Beyth S, Poradosu-Cohen R, Que YA, Beyth N, Hazan R 2015 Targeting *Enterococcus faecalis* biofilms with phage therapy. Appl Environ Microbiol 81:2696–2705. doi:10.1128/AEM.00096-15. 25662974 PMC4375334

[6] Zhang W, Mi Z, Yin X, Fan H, An X, Zhang Z, Chen J, Tong Y 2013 Characterization of *Enterococcus faecalis* phage IME-EF1 and its endolysin. PLoS One 8:e80435. doi:10.1371/journal.pone.0080435. 24236180 PMC3827423

[7] Loc-Carrillo C, Abedon ST 2011 Pros and cons of phage therapy. Bacteriophage 1:111–114. doi:10.4161/bact.1.2.14590. 22334867 PMC3278648

[8] Langenscheid J, Killmann H, Braun V 2004 A FhuA mutant of *Escherichia coli* is infected by phage T1-independent of TonB. FEMS Microbiol Lett 234:133–137. doi:10.1111/j.1574-6968.2004.tb09524.x. 15109731

[9] Yu F, Mizushima S 1982 Roles of lipopolysaccharide and outer membrane protein OmpC of *Escherichia coli* K-12 in the receptor function for bacteriophage T4. J Bacteriol 151:718–722. 7047495 10.1128/jb.151.2.718-722.1982 PMC220313

[10] Sandulache R, Prehm P, Kamp D 1984 Cell wall receptor for bacteriophage Mu G(+). J Bacteriol 160:299–303. 6384194 10.1128/jb.160.1.299-303.1984 PMC214716

[11] Marchal C, Perrin D, Hedgpeth J, Hofnung M 1980 Synthesis and maturation of lambda receptor in *Escherichia coli* K-12: *in vivo* and *in vitro* expression of gene *lamB* under *lac* promoter control. Proc Natl Acad Sci U S A 77:1491–1495. 6445557 10.1073/pnas.77.3.1491 PMC348521

[12] Baptista C, Santos MA, Sao-Jose C 2008 Phage SPP1 reversible adsorption to *Bacillus subtilis* cell wall teichoic acids accelerates virus recognition of membrane receptor YueB. J Bacteriol 190:4989–4996. doi:10.1128/JB.00349-08. 18487323 PMC2446999

[13] Tremblay DM, Tegoni M, Spinelli S, Campanacci V, Blangy S, Huyghe C, Desmyter A, Labrie S, Moineau S, Cambillau C 2006 Receptor-binding protein of *Lactococcus lactis* phages: identification and characterization of the saccharide receptor-binding site. J Bacteriol 188:2400–2410. doi:10.1128/JB.188.7.2400-2410.2006. 16547026 PMC1428394

[14] Monteville MR, Ardestani B, Geller BL 1994 Lactococcal bacteriophages require a host cell wall carbohydrate and a plasma membrane protein for adsorption and ejection of DNA. Appl Environ Microbiol 60:3204–3211. 16349376 10.1128/aem.60.9.3204-3211.1994 PMC201790

[15] Duerkop BA, Huo W, Bhardwaj P, Palmer KL, Hooper LV 2016 Molecular basis for lytic bacteriophage resistance in enterococci. mBio 7:e01304-16. doi:10.1128/mBio.01304-16. 27578757 PMC4999554

[16] Labrie SJ, Samson JE, Moineau S 2010 Bacteriophage resistance mechanisms. Nat Rev Microbiol 8:317–327. doi:10.1038/nrmicro2315. 20348932

[17] Chan BK, Sistrom M, Wertz JE, Kortright KE, Narayan D, Turner PE 2016 Phage selection restores antibiotic sensitivity in MDR *Pseudomonas aeruginosa*. Sci Rep 6:26717. doi:10.1038/srep26717. 27225966 PMC4880932

[18] Chan BK, Abedon ST, Loc-Carrillo C 2013 Phage cocktails and the future of phage therapy. Future Microbiol 8:769–783. doi:10.2217/fmb.13.47. 23701332

[19] Trotter KM, Dunny GM 1990 Mutants of *Enterococcus faecalis* deficient as recipients in mating with donors carrying pheromone-inducible plasmids. Plasmid 24:57–67. doi:10.1016/0147-619X(90)90025-8. 2125350

[20] Teng F, Singh KV, Bourgogne A, Zeng J, Murray BE 2009 Further characterization of the *epa* gene cluster and Epa polysaccharides of *Enterococcus faecalis*. Infect Immun 77:3759–3767. doi:10.1128/IAI.00149-09. 19581393 PMC2737988

[21] Theilacker C, Sava I, Sanchez-Carballo P, Bao Y, Kropec A, Grohmann E, Holst O, Huebner J 2011 Deletion of the glycosyltransferase *bgsB* of *Enterococcus faecalis* leads to a complete loss of glycolipids from the cell membrane and to impaired biofilm formation. BMC Microbiol 11:67. doi:10.1186/1471-2180-11-67. 21470413 PMC3083329

[22] Yoshida K, Yamaguchi M, Morinaga T, Kinehara M, Ikeuchi M, Ashida H, Fujita Y 2008 *myo*-Inositol catabolism in *Bacillus subtilis*. J Biol Chem 283:10415–10424. doi:10.1074/jbc.M708043200. 18310071

[23] Dale JL, Cagnazzo J, Phan CQ, Barnes AM, Dunny GM 2015 Multiple roles for *Enterococcus faecalis* glycosyltransferases in biofilm-associated antibiotic resistance, cell envelope integrity, and conjugative transfer. Antimicrob Agents Chemother 59:4094–4105. doi:10.1128/AAC.00344-15. 25918141 PMC4468649

[24] Bhardwaj P, Hans A, Ruikar K, Guan Z, Palmer KL 2018 Reduced chlorhexidine and daptomycin susceptibility in vancomycin-resistant *Enterococcus faecium* after serial chlorhexidine exposure. Antimicrob Agents Chemother 62:e01235-17. doi:10.1128/AAC.01235-17. 29038276 PMC5740357

[25] Solheim M, La Rosa SL, Mathisen T, Snipen LG, Nes IF, Brede DA 2014 Transcriptomic and functional analysis of NaCl-induced stress in *Enterococcus faecalis*. PLoS One 9:e94571. doi:10.1371/journal.pone.0094571. 24755907 PMC3995695

[26] Westra ER, van Houte S, Oyesiku-Blakemore S, Makin B, Broniewski JM, Best A, Bondy-Denomy J, Davidson A, Boots M, Buckling A 2015 Parasite exposure drives selective evolution of constitutive versus inducible defense. Curr Biol 25:1043–1049. doi:10.1016/j.cub.2015.01.065. 25772450

[27] Ainsworth S, Sadovskaya I, Vinogradov E, Courtin P, Guerardel Y, Mahony J, Grard T, Cambillau C, Chapot-Chartier MP, van Sinderen D 2014 Differences in lactococcal cell wall polysaccharide structure are major determining factors in bacteriophage sensitivity. mBio 5:e00880-14. doi:10.1128/mBio.00880-14. 24803515 PMC4010823

[28] Shibata Y, Yamashita Y, van der Ploeg JR 2009 The serotype-specific glucose side chain of rhamnose-glucose polysaccharides is essential for adsorption of bacteriophage M102 to *Streptococcus mutans*. FEMS Microbiol Lett 294:68–73. doi:10.1111/j.1574-6968.2009.01546.x. 19493010

[29] Palmer KL, Godfrey P, Griggs A, Kos VN, Zucker J, Desjardins C, Cerqueira G, Gevers D, Walker S, Wortman J, Feldgarden M, Haas B, Birren B, Gilmore MS 2012 Comparative genomics of enterococci: variation in *Enterococcus faecalis*, clade structure in *E. faecium*, and defining characteristics of *E. gallinarum* and *E. casseliflavus*. mBio 3:e00318-11. doi:10.1128/mBio.00318-11. 22354958 PMC3374389

[30] Mistou MY, Sutcliffe IC, van Sorge NM 2016 Bacterial glycobiology: rhamnose-containing cell wall polysaccharides in Gram-positive bacteria. FEMS Microbiol Rev 40:464–479. doi:10.1093/femsre/fuw006. 26975195 PMC4931226

[31] Humphries RM, Pollett S, Sakoulas G 2013 A current perspective on daptomycin for the clinical microbiologist. Clin Microbiol Rev 26:759–780. doi:10.1128/CMR.00030-13. 24092854 PMC3811228

[32] Müller A, Wenzel M, Strahl H, Grein F, Saaki TN, Kohl B, Siersma T, Bandow JE, Sahl HG, Schneider T, Hamoen LW 2016 Daptomycin inhibits cell envelope synthesis by interfering with fluid membrane microdomains. Proc Natl Acad Sci U S A 113:E7077–E7086. doi:10.1073/pnas.1611173113. 27791134 PMC5111643

[33] Dale JL, Nilson JL, Barnes AMT, Dunny GM 2017 Restructuring of *Enterococcus faecalis* biofilm architecture in response to antibiotic-induced stress. NPJ Biofilms Microbiomes 3:15. doi:10.1038/s41522-017-0023-4. 28685097 PMC5493694

[34] Kutter E, Sulakvelidze A 2005 Bacteriophages: biology and applications. CRC Press, Boca Raton, FL.

[35] Hullahalli K, Rodrigues M, Palmer KL 2017 Exploiting CRISPR-Cas to manipulate *Enterococcus faecalis* populations. Elife 6:e26664. doi:10.7554/eLife.26664. 28644125 PMC5491264

[36] Thurlow LR, Thomas VC, Hancock LE 2009 Capsular polysaccharide production in *Enterococcus faecalis* and contribution of CpsF to capsule serospecificity. J Bacteriol 191:6203–6210. doi:10.1128/JB.00592-09. 19684130 PMC2753019

[37] Cruz-Rodz AL, Gilmore MS 1990 High efficiency introduction of plasmid DNA into glycine treated *Enterococcus faecalis* by electroporation. Mol Gen Genet 224:152–154. 2126058 10.1007/BF00259462

[38] Thompson JD, Higgins DG, Gibson TJ 1994 CLUSTAL W: improving the sensitivity of progressive multiple sequence alignment through sequence weighting, position-specific gap penalties and weight matrix choice. Nucleic Acids Res 22:4673–4680. doi:10.1093/nar/22.22.4673. 7984417 PMC308517

[39] Krogh A, Larsson B, von Heijne G, Sonnhammer EL 2001 Predicting transmembrane protein topology with a hidden Markov model: Application to complete genomes. J Mol Biol 305:567–580. doi:10.1006/jmbi.2000.4315. 11152613

[40] Gold OG, Jordan HV, van Houte J 1975 The prevalence of enterococci in the human mouth and their pathogenicity in animal models. Arch Oral Biol 20:473–477. doi:10.1016/0003-9969(75)90236-8. 807189

[41] Dunny GM, Brown BL, Clewell DB 1978 Induced cell aggregation and mating in *Streptococcus faecalis*: evidence for a bacterial sex pheromone. Proc Natl Acad Sci U S A 75:3479–3483. 98769 10.1073/pnas.75.7.3479 PMC392801

[42] Leenhouts K, Buist G, Bolhuis A, ten Berge A, Kiel J, Mierau I, Dabrowska M, Venema G, Kok J 1996 A general system for generating unlabelled gene replacements in bacterial chromosomes. Mol Gen Genet 253:217–224. 9003306 10.1007/s004380050315

