# Supplementary material for: Loss-of-Function Mutations in epaR Confer Resistance to ϕNPV1 Infection in Enterococcus faecalis OG1RF
Source: Antimicrob Agents Chemother. 2018 Sep 24;62(10):e00758-18. doi: 10.1128/AAC.00758-18 (PMC6153818; doi:10.1128/AAC.00758-18)
Supplement: Supplemental file 1 [file zac010187544s1.pdf]

Table S1. Average generation times of *E. faecalis* strains.

***E. faecalis* strain**	**Average generation time (minutes)**
OG1RF	30.4
OG1RF-C	33.9
Δ*epaR*	37.4
Δ*epaR_Ew*	34.1
Δ*bgsB*	27.8
Δ*bgsB _Bw*	39.7
Δ*epaR* Δ*bgsB*	42.7

Table S2. Primers used in this study.

Number	Name	Sequence (5'-3')
1	epaR_arm1_BamHI	CGCGGATCCGCGCGATTGGTGCAACGTTGATGT
2	epaR_arm1_ovl	ACTAGCGCGGCCGCTTGCTCCTCGTCTTGCATTACTCCATTCAC
3	epaR_arm2_ovl	GGAGCAAGCGGCCGCGCTAGTGGATTGGATGAGTCTGAAGAACG
4	epaR_arm2_EcoRI	CCGGAATTCCGGTCCACTTTTCCTTTTCCTCCCT
5	EpaR_ins check for	GAAACCGCTGGGGACACATA
6	EpaR_ins check rev	TCTGGTTGGATTTCAAAATTTGAGACT
7	BgsB_arm1_BamHI	CGCGGATCCGCGGGCGTAAATAGCCATCGTATG
8	BgsB_arm1_ovl	ACTAGCGCGGCCGCTTGCTCCAGAAGTCGCTACCCCACTCA
9	BgsB_arm2_ovl	GGAGCAAGCGGCCGCGCTAGTACTTTGATCAACTTCAAATGGAAAAAG
10	BgsB_arm2_EcoRI	CCGGAATTCCGGAGGCTTTCATATAATTCTGTCGCT
11	BgsB_ins check for	ACGCTTTTATCCCTCAGAAGAA
12	BgsB_ins check rev	GGGACAAACAACACCTGTCG
13	epaR_KI for	CGCGGATCCGCGTTGGTGTCATTGGCTATCTGA
14	epaR_KI rev	CCGGAATTCCGGCGCTCATGAGTAATGTTGCCA
15	Orif	CAATAATCGCATCCGATTGCA
16	KS05	CCTATTATACCATATTTTGGA

Supplemental Figures

Figure S1

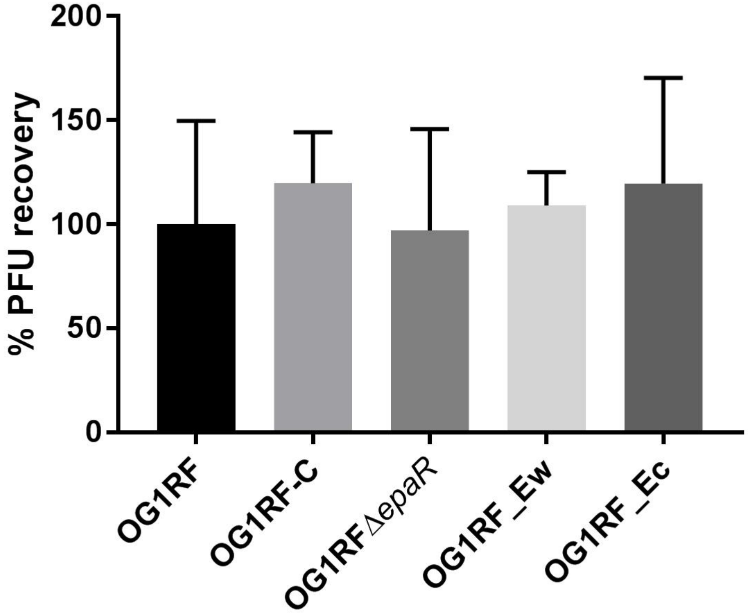

Figure S1: Percent PFU recovery from pre-incubation of phiNPV1 with polysaccharide extract. Polysaccharide extract was prepared as described above except for the final solvent being phage buffer rather than 50% acetic acid. phiNPV1 was added at a PFU of 10^5 and the mixture was incubated for 15 minutes at 37°C. The PFU was evaluated with phage spot assay. The percent PFU recovery was calculated using a phage buffer only as the base value. The experiment was performed in duplicate.

Figure S2

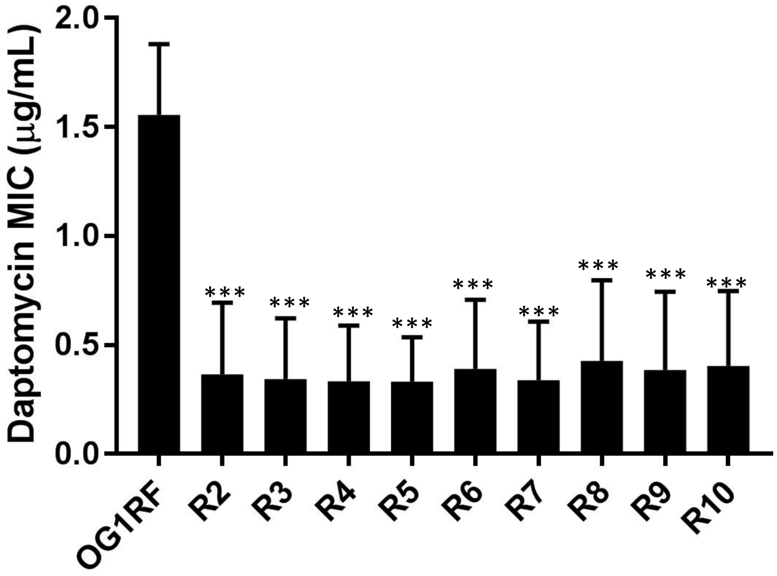

Figure S2: Daptomycin MIC of 9 phiNPV1 resistant strains derived from a confluently lysed plate. 3-5 colonies of indicated strains were resuspended in BHI and swabbed onto a MHB plate. Daptomycin strip was applied to the surface of each plate. The plates were incubated for 18 hours prior to MIC determination. The data represent the average of 6 trials. MIC data points below the detection limit of 0.016 µg/mL were taken to be 0.008 µg/mL for statistical treatment. ***, p <0.001.

Figure S3

```
EpsB      ----QFAMQDAGNNLVVLTGPTPGVGKSFVSANLAAVIATGGKRVLLIDADMRKGYLHQY     590
EpaR      DELPQLINVIRG--DMSIVGPRPERPFFVDQFN-----------------QENPNY---Y     380
WcaJ      DELPQFINVLTG--GMSIVGPRPHAVAHNEQYR-----------------QLIEGY---M     399
GumD      DELPQIFNVLGG--SMSIVGPRPHAAQHNTHYE-----------------KLINHY---M     419
WbaP      DELPQLFNVLKG--DMSLVGPRPIVSDELERYC-----------------DDVDY-----     417
CpsE      DELPQFYNVLKG--DMSLVGTRPPTVDEYEKYN-----------------STQKR-----     389
          *                  *

EpsB      FGKDRKPGLLDLLA--GNRSIEQVVHREVVPGLDFIATGLFPHNPSELLLNPRMVELMDT     648
EpaR      LRHNVRAGITGYAQVYGKYAS------------------------------------DY-N     404
WcaJ      LRHKVKPGITGWAQINGWRGET--------DTLE----------------------KM-E     428
GumD      QRHYVKPGITGWAQVNGFRGET--------PELR----------------------TM-K     448
WbaP      -YLMAKPGMTGLWQVSGRNDV------------------------------------DY-D     440
CpsE      -RLSFKPGITGLWQISGRNNIT----------------------------------DF-D     413
                    *

EpsB      FRSQYDLVLVDTPPVLAVADTAILAARAGLVLLVTRFERSTLGEIRETIKQLQHANVDVR     708
EpaR      SKLNFDLLYIKNYSLL--LDMKILLQTIKILFDKVSSRGLDESEEREEL-----------     451
WcaJ      KRVEFDLEYIREWSVW--FDIKIVFLTVFKGFVNKAAY----------------------     464
GumD      KRIQYDLDYIRRWSLW--LDIRIIVLTAVRVLGQKTAY----------------------     484
WbaP      TRVYFDSWYVKNWTLW--NDIAILFKTAKVVLRRDGAY----------------------     476
CpsE      EIVKLDVQYINEWSIW--SDIKIILLTLKVVLLGTGAK----------------------     449
           *   *
```

Figure S3: Clustal W alignment of EpaR and its homologues. Conserved residues are highlighted in red. Blue asterisks indicate where substitutions occurred in phiNPV1 resistant strains of OG1RF.
